# Home Literacy Practices and Oral Language Development of Young Immigrant Dual Language Learners: Before and During the COVID-19 Pandemic

**DOI:** 10.1017/S0305000925100147

**Published:** 2025-08-14

**Authors:** Emily Mak, Jade Lee, Qing Zhou, Yuuko Uchikoshi

**Affiliations:** 1 School of Education, University of California Davis, Davis, CA, USA; 2 Department of Psychology, University of California Berkeley, Berkeley, CA, USA

**Keywords:** home literacy practices, oral language development, dual language learners

## Abstract

This longitudinal study investigates the changes in bilingual oral language proficiency and home language and literacy practices of young Chinese American and Mexican American dual language learners (DLLs) before and during the COVID-19 pandemic. Given that DLLs represent a significant portion of young children in the U.S., often facing socioeconomic challenges, understanding their language development is crucial. The pandemic’s considerable impact on the educational trajectories of low-income immigrant communities is of particular concern. The sample comprised 132 DLLs (41 Mexican Americans and 91 Chinese Americans) from low-income immigrant families. Data on oral language proficiency in English and the home language and home literacy practices were collected before the pandemic and again 1.5 years later. Despite school closures, both groups showed improved oral language proficiency and shifts in home literacy practices over time. This study reveals distinct relationships between home literacy practices and oral language proficiency in the two immigrant groups.

## 1. Introduction

Home literacy practices for preschoolers and kindergarteners have traditionally been considered an essential stepping stone to later literacy development. However, there is little research on home literacy practices for low-income immigrant dual language learners (DLLs) in the United States. Previous literature has noted that it is essential to monitor oral language proficiency in both the home language and target language, as oral language proficiency in both languages is closely related to early literacy development (e.g., Hammer et al., 2014; Hoff, 2013; Kieffer, 2012; National Academies of Sciences, Engineering, and Medicine, 2017). Furthermore, studies show that language-focused competencies in early childhood are strongly associated with later language and literacy outcomes (Petscher et al., 2020; Shanahan & Lonigan, 2010). In particular, growing evidence emphasises the importance of supporting expanding oral language and linguistic comprehension skills (Lervåg et al., 2018). Therefore, it is crucial to understand how home literacy practices before and during the COVID-19 pandemic may have influenced DLLs’ bilingual oral language proficiency.

The significance of understanding home literacy practices is heightened when considering the demographic and socioeconomic context of DLLs in the U.S. DLLs are young children acquiring their first language and learning a second language simultaneously (Espinosa, 2013) and approximately one-third of young children (aged 0 to 5) are DLLs in the United States (Migration Policy Institute, 2021). Around one-fourth of all DLLs live in poverty, and their parents have less educational attainment than their monolingual English-speaking peers (Child Trends, 2019). Given that Chinese American children are the fastest growing and Mexican Americans are the largest immigrant population in California (Giang & Park, 2022), there is a pressing need to understand how their home literacy practices, oral language development, and other academic skills are influenced in a bilingual context.

The COVID-19 pandemic has introduced unprecedented challenges for young children. Particularly, DLL children from under-resourced contexts are expected to be most affected by the school closures due to COVID-19 (Umansky, 2020). School closures and shifts to virtual learning have drastically reduced the structured language input and interactions that DLLs typically receive in classroom settings. Also, the home environment became the primary site of language development for DLLs during the pandemic. Thus, this longitudinal study aims to determine the changes in oral proficiency and home practices of young Chinese American and Mexican American DLLs from before to during the COVID-19 pandemic. Furthermore, this study aims to understand the relationship between oral proficiency and home practices of young Chinese American and Mexican American DLLs before and during the pandemic.

## 2. Theoretical framework

The Integrative Risk and Resilience Model and the Home Literacy Model can be interconnected to offer a more comprehensive understanding of how DLLs develop their language skills and adapt to their host communities and societal disruptions like the pandemic (Suárez-Orozco et al., 2018). The Integrative Risk and Resilience Model emphasises the adaptation in the immigrant children and youths’ developmental tasks, acculturative tasks, and psychological adjustments across multiple levels, specifically global forces, political and social contexts of reception, microsystems, and individual levels. Within a microsystem, families, schools, and neighborhoods play a critical role in shaping children’s immediate experiences. During the pandemic, school closures restricted children’s interaction with their homes as the family dynamic became the primary source of language interaction. It is critical to examine these microsystem contexts because they shape and are shaped by global and social–political contexts such as attitudes and policies towards immigrants and immigration and economic inequality.

The Home Literacy Model differentiates two key ways home literacy experiences (HLEs) influence children’s later reading and writing skills (Sénéchal et al., 1998). The formal home literacy environment refers to literacy activities explicitly focused on teaching, reading, and writing skills (code-focused). These activities include teaching the alphabet, letter-sound correspondences, phonemic awareness, and word decoding. These activities are formal because they focus on building the foundational skills necessary for reading. In contrast, the informal home literacy environment includes activities that expose children to print and oral language more naturally without an explicit focus on teaching specific literacy skills (meaning-focused). These activities include shared book reading, storytelling, and casual conversations about stories. These activities are informal because the emphasis is on meaning-making and comprehension rather than decoding or word recognition. Thus, the dual-pathway model suggests that the two environments, formal and informal, influence reading outcomes differently. The Home Literacy Model proposes that formal activities enhance code-related skills, while informal activities improve oral proficiency. Multiple longitudinal studies support this model in which first language home literacy practices predicted first language oral language and literacy growth (e.g., Hood et al., 2008; Sénéchal & LeFevre, 2014; Torppa et al., 2007).

Mixed results of the Home Literacy Model have been reported among Chinese-speaking children in China versus the U.S. The findings from Zhang and Koda (2011) showed that Chinese informal literacy activities were unrelated to Chinese word knowledge among third-grade Chinese-English speakers. They measured word knowledge using tasks focused on character structure and vocabulary breadth, which are specific aspects of language. This result supports that informal literacy activities have a less direct relationship with these specific linguistic skills, and a stronger relationship with broader language development, such as listening comprehension and oral language skills. In contrast, studies conducted in China found different outcomes. Zhang and colleagues (2020) concluded that Chinese formal literacy activities in kindergarten predicted Chinese reading comprehension in first and second grade. This reflects the model’s emphasis on the role of formal literacy practices in shaping more specific literacy outcomes.

Furthermore, the Home Literacy Model was more commonly investigated in Spanish-English DLLs in the U.S. Studies concluded that the home literacy practices were associated with oral language and literacy skills in both Spanish and English (e.g., Duursma et al., 2007; Farver et al., 2013; Gonzalez & Uhing, 2008; Luo et al., 2021). Some studies also observed cross-language associations between literacy practices and language outcomes with Spanish-English bilinguals (e.g., De Ramírez & Shapiro, 2007; Farver et al., 2013; Wagley et al., 2022). Given the limited number of studies and the variability of results across linguistic contexts, it is crucial to investigate the Home Literacy Model with populations other than first-language English speakers.

Linking the Integrative Risk and Resilience Model with the Home Literacy Model helps situate home literacy practices within broader socio-political and contextual forces that shape DLL’s development. For instance, when schools were closed during the pandemic, the influence of the microsystem became more pronounced as literacy interactions within homes became even more critical for DLLs. Especially in the absence of regular school-based literacy instruction, the Home Literacy Model allows us to examine how these family-based interactions directly influence language development.

## 3. Home language and literacy practices

Home language and literacy practices encompass a wide range of language literacy activities between family members at home. These practices play a significant role in fostering children’s language and literacy development, including bilingual development (e.g., Inoue et al., 2018; Rydland & Grøver, 2021; Sénéchal et al., 1998; Sénéchal & LeFevre, 2014). For example, Luo and colleagues (2021) found that language skills mediated the relationship between literacy activities like shared reading and the language learning process in both English and Spanish. Similarly, Farver and colleagues (2013) demonstrated that home literacy practices were associated with English and Spanish oral language and pre-literacy skills among Spanish-English DLL preschoolers from low-income families. Furthermore, Durán and colleagues (2022) examined the growth rates of oral language in both English and Spanish to reveal that interactions between home language exposure and language of instruction can significantly impact language development. Gámez and colleagues (2022) found that a caregiver’s diversity of language input positively correlates with the vocabulary development of Spanish-English bilingual toddlers. Their study revealed that parents’ literacy behaviors, shared reading, and home literacy resources were linked to DLLs’ English oral language skills, whereas only parental literacy behaviors predicted Spanish oral language and literacy skills.

Studies investigating Chinese-English DLLs in the United States have shown mixed results regarding the impact of home literacy practices on Chinese literacy outcomes. While Zhang and Koda (2011) found that parents’ Chinese language use with their children was related to DLLs’ Chinese vocabulary breadth, parent–child shared reading was not found to be related to Chinese word knowledge. Instead, schoolwork-oriented literacy practices were closely related to Chinese word knowledge. These findings suggest potential differences in the effect of home literacy practices between Spanish-English and Chinese-English DLLs.

Further research has explored the connection between home literacy practices, socioeconomic status, and oral language proficiency, explicitly focusing on Spanish-English DLLs’ early literacy development (Desjardins et al., 2019; Farver et al., 2013; Luo et al., 2021; Quiroz et al., 2010). However, a critical gap remains regarding the influence of home literacy practice on English and Chinese language outcomes among low-income Chinese-English DLLs in the United States. Investigating the extent to which these practices influence oral proficiency in both the home and English languages is crucial for supporting early language and literacy development, particularly for Spanish-speaking and Chinese-speaking DLLs.

Research on the early pandemic period’s impact on home literacy practices yielded diverse results. Studies involving parent reports of monolingual English-speaking preschoolers from varied socio-economic backgrounds found no significant differences in the time spent participating in shared book reading pre-pandemic and during the pandemic (Read et al., 2021; Wheeler & Hill, 2021). However, parents reported greater distractions and reduced questioning during the pandemic during shared reading sessions. Wheeler and Hill (2021) observed that only parents who previously read to their children infrequently increased reading frequency during the pandemic, while established daily or weekly reading routines remained stable or decreased slightly. Further, Read and colleagues (2021) reported a drastic increase in screen-mediated shared reading practices despite no changes in physical book reading. Similar trends emerged in Sun and colleagues’ (2023) study of English-Mandarin Singaporean preschoolers, with increased digital media use during the first year of the pandemic. In contrast, Poulain and colleagues (2021) and Stites and colleagues (2021) observed consistent reading levels in German and American children aged 1–10 throughout the pandemic. Lin and colleagues (2023) reported decreased daily literacy activities, including book reading, writing, storytelling, and games, among English monolingual and bilingual kindergarteners during the pandemic. However, the increase in digital educational programs might compensate for the reduced schooling. On the other hand, Unsworth and colleagues (2024) found no significant differences in language use or proficiency, except for preschoolers who retained their home language despite school closures. These mixed findings suggest that the pandemic’s impact on home literacy is multifaceted and context-dependent. It may be influenced by linguistic and cultural backgrounds, adult–child interaction, and child age.

Given that DLLs represent the fastest-growing population in U.S. schools (National Center for Education Statistics, 2024), they are demonstrably the most vulnerable group and are facing language and literacy difficulties due to the loss of in-person instruction and insufficient support during the pandemic (Kuhfeld et al., 2020; Parolin & Wimer, 2020; Umansky, 2020). Additional factors, such as socio-economic or immigration status, can compound these challenges. Crosson and Silverman (2021) examined literacy instructional practices for DLLs pre-pandemic and during the pandemic. They highlighted an urgent need for focused literacy instruction during the recovery from school closures. Their study indicated a general decrease in literacy instruction due to the pandemic and emphasised the importance of prioritizing oral language and comprehension skills. In light of these findings, examining the types of home literacy practices that effectively support language development among DLLs during the pandemic is crucial.

In the current study, we broadly define the home literacy environment, drawing upon the work of Sénéchal and LeFevre (2002) and Hammer and colleagues (2003). While acknowledging that the Sénéchal and LeFevre (2002) model emphasises shared book reading and other print-rich experiences, we also consider broader aspects of home learning environments. This includes parental support for learning, which encompasses activities such as assisting with homework and providing a conducive learning environment. We recognise that parental support for learning may not perfectly align with the core components of the Sénéchal and LeFevre (2002) model, but it offers valuable insights into the broader context of children’s home learning experiences. Hence, the home literacy environment in this study encompasses shared book reading, parental assistance in learning, and storytelling.

## 4. Bilingual oral language development

Acquiring oral language proficiency is a key predictor for later literacy development and academic achievement (Hoff, 2013; NICHD Early Child Care Research Network, 2005; Storch & Whitehurst, 2002). Children who are behind on oral language proficiency are more likely to read less, miss opportunities to develop reading skills, encounter reading materials too advanced for their skills, and develop negative attitudes toward reading (Whitehurst & Lonigan, 2001; NICHD Early Child Care Research Network, 2005). Thus, creating a solid foundation for oral language proficiency during the early years is critical in literacy development.

Children’s initial exposure to language is often from interacting with parents from home environments where parents directly affect children’s learning interests (Shaffer & Kipp, 2013). Thus, children’s literacy experience at home is closely related to their reading engagement and is a prerequisite for later language development. Studies have long examined the role of the home in impacting English speakers’ oral language skills. Earlier work focused on children’s storybook exposure and early acquisition of literacy skills (Bus et al., 1995; Scarborough & Dobrich, 1994; Sénéchal et al., 1996), whereas later studies investigated home practices that include parent-to-child interactions with letters and print exposure (Burgess et al., 2002; Frijters et al., 2000; Weigel et al., 2006). In particular, the impact of language and literacy practices at home is associated with children’s oral language proficiency (Frijters et al., 2000; Sénéchal, 2006). For example, Sénéchal and Lefevre (2002) found that the parent–child reading experience impacts children’s oral and written language abilities. Li and Tan (2016) indicated that children’s language activities and literacy practices with parents contribute to their oral language ability regardless of frequency.

With the additive layer of a second language, research suggests that oral vocabulary significantly predicts reading accuracy and reading comprehension skills for DLLs (Howard et al., 2014). Research indicates that DLLs’ oral comprehension abilities in both Spanish and English are essential to their later academic success (August & Shanahan, 2006; Davison et al., 2011; Dickinson et al., 2004). Davison and colleagues (2011) and Hammer and colleagues (2007) found that Spanish-English DLL preschoolers’ oral comprehension development predicted their reading abilities at the end of kindergarten and first grade. The literature suggests two components that support DLL children’s oral comprehension ability. First, home language experience, such as exposure and the use of language, is an indicator of a DLL’s language experience at home (Bedore et al., 2012). Secondly, literacy experience, such as literacy activities and materials, offers information about the specific literacy characteristics at home (Bohman et al., 2010). It is critical to understand how these home literacy practices contribute to oral language development to support DLL children more effectively.

## 5. Current study

To address the gaps in the literature, the current study focuses on the changes in home language and literacy practices and the development of bilingual oral language proficiency of DLLs from Chinese American and Mexican American low-income immigrant families from before to during the COVID-19 pandemic. At the beginning of the COVID-19 pandemic, California recommended closure on March 19, 2020, and all schools remained closed for the remainder of the 2020–21 school year (Education Week, 2020). Most schools in California used virtual or hybrid learning at the beginning of the 2020–21 school year. By the end of the school year, only about half of the schools in California were in-person (Ballotpedia, n.d.). Due to the state school closing policies, most California children spent nearly 1.5 years in virtual or hybrid learning.

Although Spanish speakers, particularly Mexican Americans, are the majority immigrant group in the U.S., the Chinese American population has nearly doubled from 2000 to 2023 (Im, 2025), and both groups were equally represented in the Bay Area in Northern California. In addition, the relations between home literacy practices and DLLs’ bilingual oral language skills were examined for each cultural group.

We specifically sought to answer the following research questions:

1. How did the oral language proficiency in English and the home language (Spanish or Chinese) of Mexican American and Chinese American DLLs change from the pre-pandemic period to the during-pandemic period?
  We hypothesised that oral language proficiency in English and the home language would improve from the pre-pandemic period to the during-pandemic period for both cultural groups. Given the increased time children spent at home with family members who were more dominant in the home language, we anticipated observing greater growth in the home language than in English.
2. How did the home literacy practices in English and the home languages (Spanish or Chinese) of Mexican American and Chinese American DLLs change from the pre-pandemic period to the during-pandemic period?
  We hypothesised that the frequency of some home literacy practices, particularly those involving technology, would increase during the pandemic for both groups of families. Conversely, we hypothesised that the frequency of other home literacy practices, such as shared reading, would decrease during the pandemic.
3. What are the unique contributions of different home literacy practices and language input and output to the oral language development of Mexican American and Chinese American DLLs from pre-pandemic and during-pandemic?
  We hypothesised that the impact of specific home literacy practices would differ between English and the home language and that language input and output would significantly predict oral language proficiency in both languages.

## 6. Methods

In 2019, 190 DLLs (66 Mexican Americans and 124 Chinese Americans) from low-income immigrant families were recruited from Head Start programs in Northern California when they were ages three to four. To qualify for Head Start programs, families must meet federal poverty guidelines. Participation criteria included that both parents identified themselves as Chinese American or Mexican American, their home language was either Chinese (Cantonese or Mandarin) or Spanish, and the children could speak two-word phrases in their home language by age three with no developmental disorders. Informed parental consent was obtained in the parents’ preferred language at the beginning of the data collection. All assessments were conducted in one session in the child’s home by trained bilingual research assistants, and families were compensated for their time at the end of the session. Total testing time varied between 2 and 3 hours. The testing session was divided into shorter segments with multiple breaks to minimise fatigue and ensure children’s comfort.

Additionally, some tasks were designed to be more game-like to maintain engagement. We checked in with parents throughout the session to gauge their child’s comfort level and willingness to continue. If necessary, data collection was divided into two sessions over two days to accommodate the child and family’s preferences. The larger-scale study examined a broader range of factors related to language, socio-emotional development, and executive function in low-income immigrant DLL children. However, due to the specific focus of this particular paper, we only discuss measures directly relevant to the investigation of home literacy practices and child language proficiency. Children were first assessed in their dominant language, followed by their non-dominant language, by two separate trained bilingual research assistants.

Follow-up data were collected 1.5 years later during the COVID-19 pandemic from fall 2020 to fall 2021. A total of 132 families (41 Mexican Americans and 91 Chinese Americans) participated in this Time 2 follow-up data collection. Due to the COVID-19 pandemic, participating children had been out of school since March 2020. All parents were interviewed, and trained bilingual researchers assessed children over Zoom. Children were first assessed in their dominant language, followed by their non-dominant language, by two bilingual research assistants. Total testing time varied between 3 and 4 hours, and when the child became tired or unfocused, the assessments were conducted over two days. All study procedures were approved by the Committee for Protection of Human Subjects (CPHS) at the University of California, Berkeley [protocol number: 2017-06-10096].

The attrition rates for the Mexican American and Chinese American groups were 38% and 27%, respectively. There was no significant difference in per capita income or maternal educational attainment between the families who dropped out (25 Mexican Americans and 33 Chinese Americans) and those who participated in both data collection points. Table 1 presents the demographic information of the two cultural groups. Due to the significant differences in the means of children’s age at Time 2 and maternal educational years between Mexican American and Chinese American families, these two cultural groups were examined separately.

**Table 1.** Descriptive statistics of demographic information (*M* = mean, *SD* = standard deviation, Time 1 = pre-pandemic, Time 2 = during pandemic)

	Mexican American				Chinese American			
	*M*	*SD*	Min	Max	*M*	*SD*	Min	Max
Time 1 child age (month)	49.02	7.46	36	63	48.58	7.50	36	60
Time 2 child age (month)	67.19	9.18	50	88	71.27	5.89	52	88
Maternal education (year)	11.76	3.08	8	18	12.76	2.40	8	18
Family per capita income (USD)	35142.86	14513.47	5000	77500	44156.98	25022.37	5000	97500

## 7. Measures

### 7.1. Oral language proficiency

English oral language proficiency was measured by the Picture Vocabulary and Oral Comprehension subtests from the *Woodcock-Johnson, 4th Edition, Tests of Oral Language* (WJ IV OL; Schrank et al., 2014). In the Picture Vocabulary subtest, DLLs were asked to identify pictured objects and provide single-word answers. After six consecutive errors, the assessment was discontinued. The median test reliability for English at age 4 is 0.94 (McGrew et al., 2014). The Spanish version of the Picture Vocabulary subtest, Vocabulario Sobre Dibujo, was used to measure Spanish expressive vocabulary. The median test reliability for Spanish at age 4 is 0.89 (Wendling et al., 2019). Chinese American DLLs were assessed using the translated versions of the same Spanish subtest. The translations were verified by language experts (Chernoff et al., 2021), and this has been used in previous research (Chernoff et al., 2021; Chung et al., 2019; Mak et al., 2023; Uchikoshi, 2013). The alpha reliabilities in Chinese for our sample were 0.91.

In the Oral Comprehension subtest, DLLs were asked to listen to audio-recorded sentences and provide the missing words using syntactic and semantic cues. The median test reliability coefficient for English at age 4 is 0.86 (McGrew et al., 2014). The Spanish version of the Oral Comprehension subtest, Comprensión Oral, assessed Spanish oral language. The median test reliability for Spanish at age 4 is 0.91 (Schrank et al., 2005). Chinese American DLLs were assessed using the translated versions of the same Spanish subtest. The alpha reliability for this sample in Chinese was 0.87. Raw scores were used in data analyses since we did not have standardised scores for home language proficiency for the Chinese American DLLs.

To mitigate the potential challenges associated with remote testing at Time 2, we ensured participants had stable internet connections and provided tablets for testing if needed. Our research team was also available to provide technical assistance via phone during the testing session. We adhered to the standardised testing protocols to ensure consistency across participants. In addition, we supplemented the oral language tasks with visual aids and interactive elements, such as using animated red boxes to indicate the picture being referred to for Picture Vocabulary, to enhance engagement and provide additional context for the children. While there may be some limitations to remote testing compared to in-person assessments, these adaptations helped maintain the validity and reliability of the data collected at Time 2.

### 7.2. Home literacy practices

Parents were asked to complete a questionnaire to report their demographic information and English and home language literacy practices used in past research (e.g., Haft et al., 2021). Parents were given a choice of filling out the questionnaires in English, Spanish, or Chinese (traditional and simplified). The assessment of HLE drew upon the frameworks of Sénéchal and LeFevre (2002) and Hammer and colleagues (2003). The home literacy activities in English and the home language were adapted from Hammer and colleagues (2003). Parents reported (a) parent–child shared book reading frequency (0 = *never*, 1 = *once a month*, 2 = *2–4 times a month*, 3 = *once a week*, 4 = *2–3 times a week*, 5 = *every day*), (b) help received for learning (0 = *never*, 1 = *once a month*, 2 = *2–3 times a month*, 3 = *1–2 times a week*, 4 = *every day*), (c) oral storytelling by adults at home (0 = *never*, 1 = *once a month*, 2 = *2–3 times a month*, 3 = *1–2 times a week*, 4 = *every day*).

### 7.3. Media exposure

Parents also reported the number of hours their child was exposed to media in English and the home language on a typical day.

### 7.4. Bilingual input and output

The questions consisted of hour-by-hour child language input and output adapted from the Bilingual Input–Output Survey (BIOS; Peña et al., 2014). Parents reported on the child’s time of at-home language exposure during a typical week and the time of at-home language output during a typical week.

## 8. Data analysis

First, all variables were screened for normality using the Shapiro–Wilk tests. Friedman tests were used to examine significant differences between Time 1 (pre-pandemic) and Time 2 (during the pandemic) for the non-normally distributed variables for the Mexican American and Chinese American groups separately. Kendall’s correlations were calculated to indicate the associations between pre-pandemic and during-pandemic variables. These correlations were exploratory in examining the potential relationships between multiple variables, including independent home literacy variables and vocabulary, and correlations within the home literacy variables. No corrections for multiple correlations were applied, which warrants cautious interpretations. Further, the data for home literacy practices were examined and evaluated with principal component analysis. Finally, based on the results of the correlations and Principal Component Analysis, multiple regression analyses were performed to examine the relationships between home literacy practices, bilingual input and output, and oral language proficiency for each cultural group. All data analyses were conducted in RStudio Version 2023.12.1 + 402 (RStudio Team, 2021).

## 9. Results

The descriptive statistics of the oral language variables for Mexican American and Chinese American participants are displayed in Tables 2 and 3, respectively. Standard scores of both the Picture Vocabulary and Oral Comprehension subtests reveal that both groups scored 1.5 to 2 standard deviations below the published age-matched monolingual norms in English before and during the pandemic. Additionally, the standard scores for both subtests in Spanish were over two standard deviations below the published age-matched monolingual norms before and during the pandemic for the Spanish-speaking DLLs. Since the study variables were not normally distributed, the Friedman non-parametric test, equivalent to repeated measures ANOVA, was computed. The Friedman tests showed that there were significant increases from pre-pandemic to during the pandemic among the Mexican American DLLs in their Picture Vocabulary scores in English, *χ* ^2^ (1) = 22.73, *p* < .001, and Spanish, *χ* ^2^ (1) = 23.06, *p* < .001. Similar results were observed in the Chinese Americans with significant increases in their Picture Vocabulary scores in English, *χ* ^2^ (1) = 79.05, *p* < .001, and in Chinese, *χ* ^2^ (1) = 27.96, *p* < .001. Additionally, the scores of Oral Comprehension for the Mexican American group increased from pre-pandemic to during the pandemic in English, *χ* ^2^ (1) = 30.12, *p* < .001, and Spanish, *χ* ^2^ (1) = 31.00, *p* < .001. The scores of Oral Comprehension for the Chinese American group also increased from pre-pandemic to during the pandemic in English, *χ* ^2^ (1) = 74.20, *p* < .001, and Chinese, *χ* ^2^ (1) = 47.52, *p* < .001.

**Table 2.** Descriptive statistics of oral language proficiency variables pre-pandemic and during pandemic for Mexican American children (*M* = mean, *SD* = standard deviation, PV = Woodcock Johnson Picture Vocabulary, OC = Woodcock Johnson Oral Comprehension)

	Pre-pandemic							During pandemic						
	*N*	*M*	*SD*	Min	Max	Skewness	Kurtosis	*N*	*M*	*SD*	Min	Max	Skewness	Kurtosis
Child age (months)	41	49.02	7.46	36	63	−0.06	−1.37	39	67.18	9.18	50	88	0.11	−0.32
English PV (standard score)	41	76.56	19.08	40	117	−0.18	−0.84	34	78.03	15.50	40	110	−0.30	−0.30
Spanish PV (raw score)	40	10.23	6.97	1	22	0.42	−1.25	37	15.35	8.37	2	30	−0.07	−1.21
English OC (standard score)	41	73.93	15.07	51	118	0.83	0.36	35	82.57	17.99	52	119	0.04	−1.03
Spanish OC (raw score)	41	1.34	1.94	0	8	1.76	2.73	38	6.50	5.22	0	17	0.55	−0.84

**Table 3.** Descriptive statistics of oral language proficiency variables pre-pandemic and during pandemic for Chinese American children (*M* = mean, *SD* = standard deviation, PV = Woodcock Johnson Picture Vocabulary, OC = Woodcock Johnson Oral Comprehension)

	Pre-pandemic							During pandemic						
	*N*	*M*	*SD*	Min	Max	Skewness	Kurtosis	*N*	*M*	*SD*	Min	Max	Skewness	Kurtosis
Child age (months)	91	48.58	7.50	36	60	−0.16	−1.22	89	71.27	5.89	52	88	−0.19	1.11
English PV (standard score)	86	78.95	15.44	40	114	−0.37	−0.41	85	89.27	13.67	45	120	−0.30	−0.30
Chinese PV (raw score)	90	10.66	6.70	0	23	0.06	−1.35	86	14.31	6.21	0	27	−0.48	−0.14
English OC (standard score)	86	70.05	14.56	49	116	0.83	0.36	85	89.61	17.42	40	121	0.04	−1.03
Chinese OC (raw score)	87	1.15	1.97	0	12	2.68	9.75	84	4.26	3.48	0	12	0.53	−0.71

The descriptive statistics of the home literacy practice variables for Mexican American participants are shown in Table 4. There were no significant changes in the Mexican American DLLs’ home literacy practices from pre-pandemic to during the pandemic. On average, the parents of Mexican American DLLs spent roughly 2–4 times a month reading to their DLL children in English before and during the pandemic. On the other hand, they spent more than once a week on average reading to the children in Spanish before and during the pandemic. In addition, the Mexican American parents helped their children learn in both English and Spanish about once a week on average before and during the pandemic. These parents also told stories to their children in English on average for about 2–3 times a month and in Spanish close to 1–2 times a week. On average, the Spanish-English DLL children were exposed to English media 1.29 and 1.43 hours daily before and during the pandemic, respectively. They were exposed to Spanish media 0.97 and 1.15 hours on average every day before and during the pandemic, respectively. On average, the proportion of English language input of the Mexican American DLLs increased significantly from 46% pre-pandemic to 52% during the pandemic. In comparison, Spanish language input decreased from 62% to 51%, *χ* ^2^ (1) = 5.76, *p* < .05. In terms of language output, there was a significant increase in English language output for Mexican American children from an average of 46% of the time pre-pandemic to 52% of the time during the pandemic, while the proportion of time they spoke in Spanish decreased from 54% pre-pandemic to 48% during the pandemic, *χ* ^2^ (1) = 5.76, *p* < .05.

**Table 4.** Descriptive statistics of home literacy practice variables pre-pandemic and during pandemic for Mexican American children (M = mean, *SD* = standard deviation)

	Pre-pandemic							During pandemic						
	*N*	*M*	*SD*	Min	Max	Skewness	Kurtosis	*N*	*M*	*SD*	Min	Max	Skewness	Kurtosis
English reading	37	2.35	1.93	0	5	−0.13	−1.71	41	2.71	1.89	0	5	−0.41	−1.41
Spanish reading	37	3.57	1.50	0	5	−1.18	0.35	41	3.39	1.69	0	5	−1.04	−0.26
English learning	38	2.97	1.37	0	4	−1.26	0.24	41	3.34	1.06	0	4	−1.54	1.38
Spanish learning	37	2.97	1.24	0	4	−1.41	0.99	41	2.42	1.61	0	4	−0.46	−1.48
English storytelling	38	2.29	1.52	0	4	−0.44	−1.33	41	2.07	1.62	0	4	−0.22	−1.62
Spanish storytelling	34	2.68	1.47	0	4	−0.89	−0.74	41	2.63	1.39	0	4	−0.81	−0.68
English media exposure	39	1.29	0.81	0	3	0.16	−1.00	40	1.43	1.33	0	8	3.01	12.24
Spanish media exposure	39	0.97	0.86	0	3	0.43	−1.00	38	1.15	0.96	0	4	1.03	0.63
English input	38	0.38	0.21	0	0.81	0.14	−0.82	39	0.49	0.24	0	1	0.03	−0.38
Spanish input	38	0.62	0.21	0.19	1	−0.14	−0.82	39	0.51	0.24	0	1	−0.03	−0.38
English output	38	0.46	0.28	0	1	0.43	−0.62	39	0.52	0.27	0	1	0.10	−0.59
Spanish output	38	0.54	0.28	0	1	−0.43	−0.62	39	0.48	0.27	0	1	−0.10	−0.59

The descriptive statistics of the home literacy practice variables for Chinese American participants are shown in Table 5. There were changes in the average frequency of certain home literacy activities in both English and Chinese. On average, the Chinese American parents assisted their DLL children’s learning in English more frequently during the pandemic (once a week) than pre-pandemic (2–4 times a month), *χ* ^2^ (1) = 15.68, *p* < .001. Additionally, the Chinese American DLLs were generally exposed to English media more often during the pandemic than pre-pandemic, *χ* ^2^ (1) = 28.52, *p* < .001. In terms of Chinese literacy activities, the Chinese American parents spent over once a month telling stories in Chinese during the pandemic, which was less than pre-pandemic when they told stories 2–4 times a month, *χ* ^2^ (1) = 4.41, *p* < .05. Furthermore, they used to read Chinese books with their children once a week before the pandemic while they only read 2–4 times a month during the pandemic, *χ* ^2^ (1) = 5.92, *p* < .05. Before the pandemic, the parents on average spoke Chinese 61% of the time and English 40% of the time. However, they spoke more English (52%) than Chinese (48%) during the pandemic. The Chinese-English DLLs used to speak in Chinese 57% of the time and English 43% of the time before the pandemic, but they started spending 55% of the time speaking in English rather than in Chinese during the pandemic, *χ* ^2^ (1) = 8.38, *p* < .01.

**Table 5.** Descriptive statistics of home literacy practice variables pre-pandemic and during pandemic for Chinese American children (*M* = mean, *SD* = standard deviation)

	Pre-pandemic							During pandemic						
	*N*	*M*	*SD*	Min	Max	Skewness	Kurtosis	*N*	*M*	*SD*	Min	Max	Skewness	Kurtosis
English reading	90	2.10	2.01	0	5	0.16	−1.69	91	2.36	2.07	0	5	−0.01	−1.68
Chinese reading	90	3.00	1.87	0	5	−0.56	−1.23	91	2.41	1.87	0	5	−0.08	−1.56
English learning	90	2.14	1.65	0	4	−0.30	−1.63	91	2.85	1.58	0	4	−0.93	−0.60
Chinese learning	90	2.50	1.57	0	4	−0.64	−1.22	90	2.81	1.44	0	4	−0.93	−0.06
English storytelling	90	1.90	1.58	0	4	−0.07	−1.64	91	2.14	1.61	0	4	−0.28	−1.56
Chinese storytelling	90	2.33	1.55	0	4	−0.47	−1.36	91	1.95	1.36	0	4	−0.08	−1.30
English media	90	1.23	0.93	0	6	1.74	6.06	91	2.04	1.52	0	10	1.95	6.89
Chinese media	89	0.42	0.55	0	2.50	0.14	−0.84	90	0.56	1.24	0	10	5.42	35.99
English input	90	0.40	0.17	0.11	0.80	0.14	−0.84	89	0.52	0.17	0.08	0.95	0.05	−0.12
Chinese input	90	0.61	0.17	0.20	0.89	−0.14	−0.84	89	0.48	0.17	0.05	0.92	−0.05	−0.13
English output	90	0.43	0.22	0	1	0.24	−0.26	89	0.55	0.19	0	1	0	0.46
Chinese output	90	0.57	0.22	0	1	−0.24	−0.26	89	0.45	0.19	0	1	0	0.46

### 9.1. Correlations

Kendall’s correlations were performed to explore the associations between pre-pandemic variables, during-pandemic variables, and oral language proficiency outcome variables. Table 6 presents the correlation results of the Time 1 pre-pandemic variables for the Mexican American DLLs. Their English Picture Vocabulary standard score was positively correlated with English media exposure (*r* = .27, *p* < .05), English language input (*r* = .21, *p* < .05), and English language output (*r* = .29, *p* < .05) in English. English Oral Comprehension standard score was positively correlated with shared book reading frequency in English (*r* = .33, *p* < .01). Spanish Picture Vocabulary raw score was moderately correlated with shared book reading (*r* = .44, *p* < .01), storytelling (*r* = .38, *p* < .05), and language output (*r* = .28, *p* < .01) in Spanish. Similarly, Spanish Oral Comprehension raw score was positively correlated with Spanish shared book reading (*r* = .37, *p* < .05), Spanish storytelling (*r* = .37, *p* < .05), and Spanish output (*r* = .34, *p* < .05).

**Table 6.** Correlations of pre-pandemic study variables for Mexican American children (ENG = English, SPA = Spanish, PV = Woodcock Johnson Picture Vocabulary, OC = Woodcock Johnson Oral Comprehension, R = raw score, S = standard score. *p < .05, ^**^p < .01, ^***^p < .001)

Variable	1	2	3	4	5	6	7	8	9	10	11	12	13	14	15	16
ENG PV (S)	–															
ENG OC (S)	.24^**^	–														
SPA PV (R)	−.16	.15	–													
SPA OC (R)	−.08	.13	.64^***^	–												
ENG reading	.17	.33^**^	.02	−.02	–											
SPA reading	−.01	.14	.44^**^	.37*	.10	–										
ENG learning	.12	−.01	−.17	−.20*	.26*	−.20	–									
SPA learning	−.11	.01	.19	.17	.01	.28	.41^**^	–								
ENG storytelling	.22	.03	−.11	−.12	.10	−.05	.36^**^	.02	–							
SPA storytelling	−.28	.21	.38*	.37*	.07	.51^**^	−.34*	.19	−.21	–						
ENG media	.27*	−.03	−.30*	−.37^***^	.06	−.29*	−.32*	−.13	.44^**^	−.32*	–					
SPA media	.24	.21	.08	.06	−.03	.11	−.05	.11	−.05	.11	.06	–				
ENG input	.22*	−.02	−.32*	−.30	.02	−.31*	.12	−.18	.30^**^	−.42^***^	.28*	−.32^**^	–			
SPA input	−.22*	.02	.32	.30	−.02	.31*	−.12	.18	−.30^**^	.42^***^	−.28*	.32^**^	−1^***^	–		
ENG output	.29*	−.19	−.28^**^	−.34*	.04	−.36^**^	.08	−.30*	.27*	−.54^***^	.29*	−.18	.63^***^	−.63^***^	–	
SPA output	−.29*	.19	.28^**^	.34*	.04	.36^**^	−.08	.30*	−.27*	.54^***^	−.29*	.18	−.63^***^	.63^***^	−1^***^	–

Table 7 shows the correlation results for the Time 2 during-pandemic variables for the Mexican American DLLs. Their English Picture Vocabulary raw score was positively correlated with English storytelling (*r* = .25, *p* < .05), English language input (*r* = .28, *p* < .05), and English language output (*r* = .32, *p* < .001). Spanish Picture Vocabulary raw score was correlated with help received in Spanish learning (*r* = .35, *p* < .01) and Spanish language output (*r* = .25, *p* < .05).

**Table 7.** Correlations of during pandemic study variables for Mexican American children (ENG = English, SPA = Spanish, PV = Woodcock Johnson Picture Vocabulary, OC = Woodcock Johnson Oral Comprehension, R = raw score, S = standard score. *p < .05, ^**^p < .01, ^***^p < .001)

Variable	1	2	3	4	5	6	7	8	9	10	11	12	13	14	15	16
ENG PV (S)	–															
ENG OC (S)	.55^***^	–														
SPA PV (R)	−.08	.21	–													
SPA OC (R)	−.10	.21	.68^***^	–												
ENG reading	.19	.13	−.25*	−.16	–											
SPA reading	.03	.09	.17	.20	−.17	–										
ENG learning	.07	.04	.05	−.05	.24	.02	–									
SPA learning	.08	.12	.35^**^	.27	−.10	.21	.12	–								
ENG storytelling	.35*	−.03	−.14	−.24*	.28*	−.21	.20	.18	–							
SPA storytelling	.05	.08	.14	.12	.17	.26	0.00	.23*	.21	–						
ENG media	.09	−.05	−.21*	−.24*	.19	−.02	.10	−.04	.22	.23	–					
SPA media	−.10	−.01	.23	.22	−.19	.29*	−.16	.28	0.00	.42^**^	.16	–				
ENG input	.23*	.09	−.21	−.14	.20	−.25*	.14	−.13	.20	−.32^**^	.14*	−.19	–			
SPA input	−.23*	−.09	.21	.14	−.20	.25*	−.14	.13	−.20	.32^**^	−.14*	.19	−1^***^	–		
ENG output	.23^**^	.05	−.25*	−.25	.19	−.28*	.13	−.22*	.20	−.29^**^	.07*	−.34^**^	.78^***^	−.78^***^	–	
SPA output	−.23^**^	−.05	.25*	.25	−.19	.28*	−.13	.22*	−.21	.29^**^	−.07*	.34^**^	−.78^***^	.78^***^	−1^***^	–

Tables 8 and 9 display the correlation results of the Chinese American DLLs’ Time 1 pre-pandemic and Time 2 during-pandemic variables, respectively. Before the pandemic, their English Picture Vocabulary standard score was correlated with child English language output (*r* = .26, *p* < .05). English Oral Comprehension standard score was correlated with English shared reading (*r* = .28, *p* < .01), and English storytelling (*r* = .25, *p* < .05). Chinese Picture Vocabulary raw score was correlated with Chinese shared reading (*r* = .27, *p* < .05), help received with Chinese learning (*r* = .27, *p* < .05), Chinese storytelling (*r* = .30, *p* < .01), Chinese media exposure (*r* = .25, *p* < .05), Chinese language input (*r* = .41, *p* < .001), and Chinese language output (*r* = .42, *p* < .001). Chinese Oral Comprehension raw score was correlated with Chinese media exposure (*r* = .24, *p* < .05). During the pandemic, Chinese American DLLs’ English Picture Vocabulary raw score was correlated with English media exposure (*r* = .21, *p* < .05). Their English Oral Comprehension raw score was correlated with help received for learning in English (*r* = .23, *p* < .05) and English storytelling (*r* = .38, *p* < .001).

**Table 8.** Correlations of pre-pandemic study variables for Chinese American children (ENG = English, CHI = Chinese, PV = Woodcock Johnson Picture Vocabulary, OC = Woodcock Johnson Oral Comprehension, R = raw score, S = standard score. *p < .05, ^**^p < .01, ^***^p < .001)

Variable	1	2	3	4	5	6	7	8	9	10	11	12	13	14	15	16
ENG PV (S)	–															
ENG OC (S)	.39^***^	–														
CHI PV (R)	−.29^**^	−.03	–													
CHI OC (R)	.09	.27*	.48^***^	–												
ENG reading	.21	.28^**^	−.05	.10	–											
CHI reading	−.12	−.02	.27*	.08	.24*	–										
ENG learning	.04	.04	.09	.04	.36^***^	.04	–									
CHI learning	−.13	−.19	.27*	.13	.08	.25*	.24*	–								
ENG storytelling	.20	.25*	−.04	.04	.64^***^	.23*	.54^***^	.16	–							
CHI storytelling	−.20	−.18	.30^**^	.06	.06	.75^***^	0	.32^**^	.11	–						
ENG media	.12	.03	−.33^**^	−.18	−.20	−.30^**^	−.01	−.25*	−.21*	−.29^**^	–					
CHI media	−.06	.08	.25*	.24*	.02	.01	−.14	.11	−.14	−.07	−.16	–				
ENG input	.18	.06	−.41^***^	−.13	.13	−.25*	.18	−.16	.09	−.38^***^	.29^**^	−.19	–			
CHI input	−.18	−.06	.41^***^	.13	−.13	.25*	−.18	.16	−.09	.38^***^	−.29^**^	.19	−1^***^	–		
ENG output	.26*	.03	−.42^***^	−.15	.17	−.22*	.12	−.10	.14	−.31^**^	.22*	−.15	.83^***^	−.83^***^	–	
CHI output	−.26*	−.03	.42^***^	.15	−.17	.22*	−.12	.10	−.14	.31^**^	−.22*	.15	−.83^***^	.83^***^	−1^***^	–

**Table 9.** Correlations of during pandemic study variables for Chinese American children (ENG = English, CHI = Chinese, PV = Woodcock Johnson Picture Vocabulary, OC = Woodcock Johnson Oral Comprehension, R = raw score, S = standard score. *p < .05, ^**^p < .01, ^***^p < .001)

Variable	1	2	3	4	5	6	7	8	9	10	11	12	13	14	15	16
ENG PV (S)	–															
ENG OC (S)	.59^***^	–														
CHI PV (R)	−.27*	−.03	–													
CHI OC (R)	−.24*	.05	.67^***^	–												
ENG reading	.10	.20	.08	−.04	–											
CHI reading	−.12	.18	.43^***^	.28*	.23*	–										
ENG learning	.18	.23*	.01	−.10	.49^***^	.16	–									
CHI learning	−.23*	−.11	.11	.17	−.08	.09	−.04	–								
ENG storytelling	.16	.38^***^	.04	.04	.60^***^	.36^***^	.53^***^	.05	–							
CHI storytelling	−.06	.13	.38^***^	.09	.03	.63^***^	.01	.21	.25*	–						
ENG media	.21*	.05	−.15	−.15	0	−.23*	−.03	−.18	−.19	−.26*	–					
CHI media	−.10	.06	.28^**^	.31^**^	.08	.03	−.05	−.04	.02	−.06	.41^***^	–				
ENG input	−.11	−.19	.06	−.13	.08	.03	−.01	.14	−.07	.18	−.04	−.08	–			
CHI input	.11	.19	−.06	.13	−.08	−.03	.01	−.14	.06	−.18	.04	.08	−1^***^	–		
ENG output	.05	−.03	−.04	−.24*	.02	.06	−.04	.04	−.06	.14	−.08	−.05	.67^***^	−.67^***^	–	
CHI output	−.05	.03	.04	.24*	−.02	−.06	.04	−.04	.06	−.14	.08	.05	−.67^***^	.67^***^	−1^***^	–

### 9.2. Principal component analysis

Home literacy practices were measured with three items: reading, learning, and storytelling. The mean values of these variables measured before and during the pandemic are stated in Table 4 for the Mexican American group and Table 5 for the Chinese American group. As the literacy practices were on different scales, the scores were first standardised. Then, the factorability of the home literacy practices for each language at each time point was examined separately for the Mexican and Chinese Americans. Cronbach’s alpha for the three English literacy practices at Time 1 was 0.73. Cronbach’s alpha for the three home language literacy practices at Time 1 was 0.69. At Time 2, all three English literacy practices were significantly correlated. Cronbach’s alpha for the three English literacy practices at Time 2 was 0.73. For the home language, only reading and storytelling practices were significantly correlated. Cronbach’s alpha for these two home language literacy practices at Time 2 was 0.72.

For pre-pandemic (Time 1) home literacy practices, principal component analysis with varimax rotation on the three English home literacy practices extracted one component with an Eigenvalue higher than 1 for Mexican American DLLs. The Kaiser–Meyer–Olkin measure of sampling adequacy was 0.55, higher than the recommended value of 0.5. The Barlett’s Test of Sphericity was significant, *χ* ^2^ (3) = 15.15, *p* < .01. All three items (i.e., reading, learning, and storytelling) loaded on one component, “Time 1 English Home Literacy.” For the home language, principal component analysis with varimax rotation on the three home literacy practices indicated one component with an Eigenvalue higher than 1. The Kaiser–Meyer–Olkin measure of sampling adequacy was 0.55. The Barlett’s Test of Sphericity was significant, *χ* ^2^ (3) = 12.27, *p* < .01. All three items loaded on one component, “Time 1 Spanish Home Literacy.”

For the Chinese American DLLs, principal component analysis with varimax rotation on the three English home literacy practices indicated one component with an Eigenvalue higher than 1. The Kaiser–Meyer–Olkin measure of sampling adequacy was 0.62. The Barlett’s Test of Sphericity was significant, *χ* ^2^ (3) = 76.23, *p* < .001. All three items loaded on one component, “Time 1 English Home Literacy.” For the home language, principal component analysis with varimax rotation on the three home literacy items indicated one component with an Eigenvalue higher than 1. The Kaiser–Meyer–Olkin measure of sampling adequacy was 0.56. The Barlett’s test of sphericity was significant, *χ* ^2^ (3) = 80.73, *p* < .001. Three items were loaded on the component “Time 1 Chinese Home Literacy.”

Regarding during-pandemic (Time 2) home literacy practices, for Mexican American DLLs, principal component analysis with varimax rotation on the three English home literacy practices extracted one component with an Eigenvalue higher than 1. The Kaiser–Meyer–Olkin measure of sampling adequacy was 0.60. Barlett’s test of sphericity was marginally significant, *χ* ^2^ (3) = 8.05, *p* < .05. English reading, learning, and storytelling loaded on one component, “Time 2 English Home Literacy.” Regarding Spanish home literacy practices during the pandemic, the variables did not load well onto the principal components.

For Chinese American DLLs, principal component analysis with varimax rotation on the three home literacy practices extracted two components with an Eigenvalue higher than 1. The Kaiser–Meyer–Olkin measure of sampling adequacy was 0.69. The Barlett’s test of sphericity was significant, *χ* ^2^ (3) = 75.22, *p* < .001. English reading, storytelling, and learning were loaded on one component, “Time 2 English Home Literacy.” Regarding Chinese home literacy practices during the pandemic, principal component analysis with varimax rotation on the reading and storytelling extracted one component with an Eigenvalue higher than 1. The Kaiser–Meyer–Olkin measure of sampling adequacy was 0.50. The Barlett’s test of sphericity was significant, *χ* ^2^ (1) = 45.48, *p* < .001. Two items (home language reading and storytelling) loaded on one component, “Time 2 Chinese Home Literacy.”

### 9.3. Multiple regression analysis

**Figure 1.**
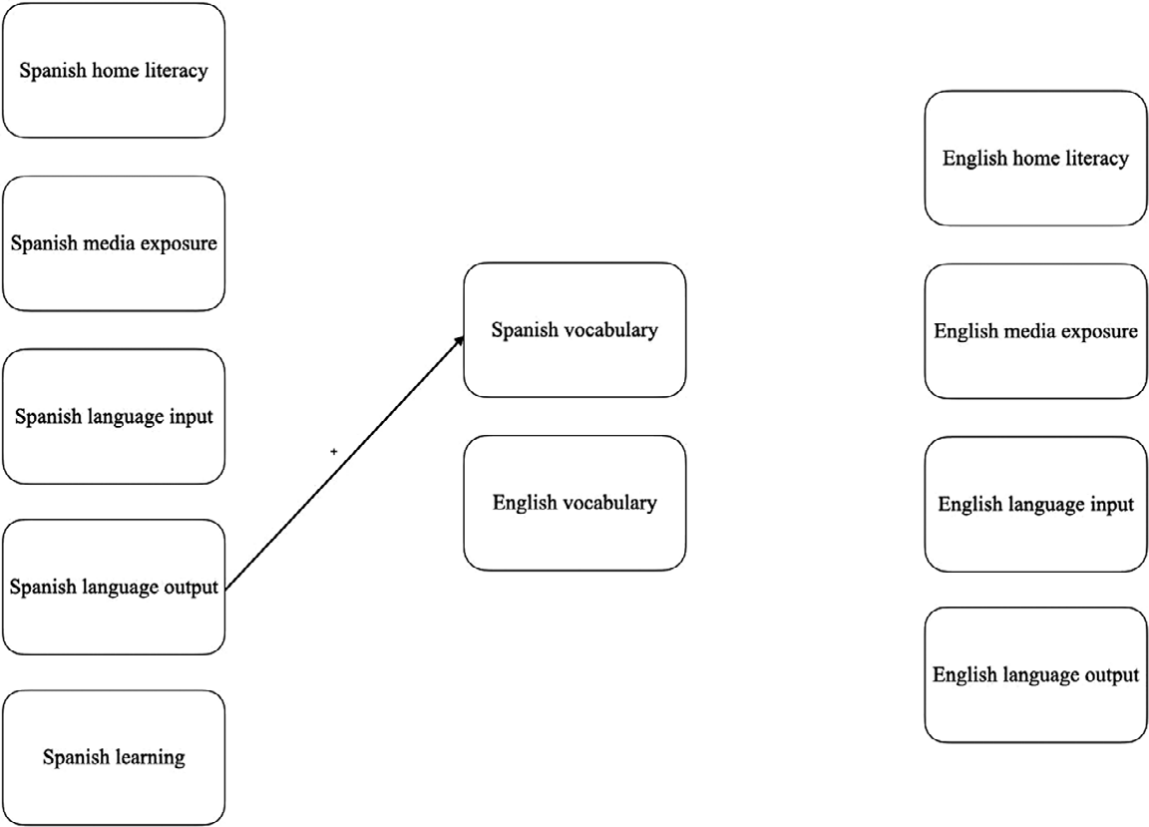
Relationships between Home Literacy Practices, Language Input/Output, and Vocabulary for Mexican American Participants Pre-Pandemic and During the Pandemic. Solid line = Time 1 and Time 2 significant relationships, + = positive relationship. Vocabulary was measured with Picture Vocabulary and Oral Comprehension. Child age and pre-pandemic vocabulary were controlled. Standardised beta coefficients can be found in the multiple regression tables.

Multiple regression analyses were conducted to predict oral language proficiency using the components of home literacy practices, media exposure, and bilingual output and input by cultural groups at two time points. As shown in Table 10, Mexican American DLLs’ Spanish language output was positively associated with Spanish Picture Vocabulary raw score pre-pandemic. Spanish language output was significantly related to both Spanish Picture Vocabulary and oral comprehension raw scores during the pandemic after controlling for pre-pandemic Spanish oral proficiency scores and child age. For the Mexican American DLLs’ oral language proficiency in English (Table 11), the regression models were not significant in predicting English picture vocabulary or oral comprehension standard scores before or during the pandemic. These relationships are visually summarised in Figure 1.

**Table 10.** Multiple regression testing relations of Spanish Home Literacy Practices and Spanish Oral Language Proficiency in Mexican American children (T1 = Time 1 [pre-pandemic], T2 = Time 2 [during pandemic], SPA = Spanish, PV = Woodcock Johnson Picture Vocabulary, OC = Woodcock Johnson Oral Comprehension, R = raw score, *B* = unstandardised regression coefficient, *β* = standardised regression coefficient, SE = standard error; *p < .05, ^***^p < .001)

	T1 SPA PV (R)			T1 SPA OC (R)			T2 SPA PV (R)			T2 SPA OC (R)		
Predictors	*B*	*β*	SE	*B*	*β*	SE	*B*	*β*	SE	*B*	*β*	SE
T1 SPA home literacy	2.26	.31	1.26	.55	.26	.46						
T1 SPA media	.69	.08	1.36	.62	.24	.50						
T1 SPA input	−4.77	−.14	6.80	−3.10	−.32	2.47						
T1 SPA output	14.74*	.53	6.23	4.20	.53	2.26						
T2 SPA learning							1.07	.21	.59	.09	.03	.42
T2 SPA input							−8.14	−.23	7.34	−9.13	−.43	5.26
T2 SPA output							15.97*	.50	6.85	14.62*	.76	4.94
T1 child age	.57^***^	.59	.14	.11*	.40	.05						
T2 child age							.22	.23	.11	.15	.26	.08
T1 SPA PV (R)							.71^***^	.58	.14			
T1 SPA OC (R)										1.51^***^	.53	.37
Constant	−21.46*		7.86	−4.68		2.86	−13.04		8.26	−8.69		5.90
*R^2^*	.60			.36			.67			.53		
Adjusted *R^2^*	.52			.23			.62			.46		
*F*	7.18^***^			2.71*			12.40^***^			6.89^***^		
*N*	31			31			36			36

**Table 11.** Multiple regression testing relations of English Home Literacy Practices and English Oral Language Proficiency in Mexican American children (T1 = Time 1 [pre-pandemic], T2 = Time 2 [during pandemic], ENG = English, PV = Woodcock Johnson Picture Vocabulary, OC = Woodcock Johnson Oral Comprehension, S = standard score, HLP = home literacy practices, *B* = unstandardised regression coefficient, *β* = standardised regression coefficient, SE = standard error; *p < .05, ^***^p < .001)

	T1 ENG PV (S)			T1 ENG OC (S)			T2 ENG PV (S)			T2 ENG OC (S)		
Predictors	*B*	*β*	SE	*B*	*β*	SE	*B*	*β*	SE	*B*	*β*	SE
T1 ENG home literacy	1.84	.10	3.28	3.96	.27	2.78						
T1 ENG media	4.27	.18	4.63	−.81	−.04	3.91						
T1 ENG input	11.46	.13	19.13	21.60	.30	16.17						
T1 ENG output	14.42	.21	15.13	−15.08	−.27	12.79						
T2 ENG home literacy							2.05	.13	3.10	.33	.02	3.77
T2 ENG media							.15	.01	2.00	−3.63	−.28	2.46
T2 ENG input							−19.41	−.31	25.30	21.98	.29	30.84
T2 ENG output							31.96	.55	23.51	−5.03	−.07	28.94
T1 ENG PV (S)							.25	.30	.15			
T1 ENG OC (S)										.49*	.43	.20
Constant	62.87^***^		7.44	76.00^***^		6.29	52.14^***^		11.68	43.36*		16.71
*R^2^*	.21			.14			.33			.29		
Adjusted *R^2^*	.11			.03			.20			.15		
*F*	2.00			1.22			2.58			2.13		
*N*	37			37			36			38

**Figure 2.**
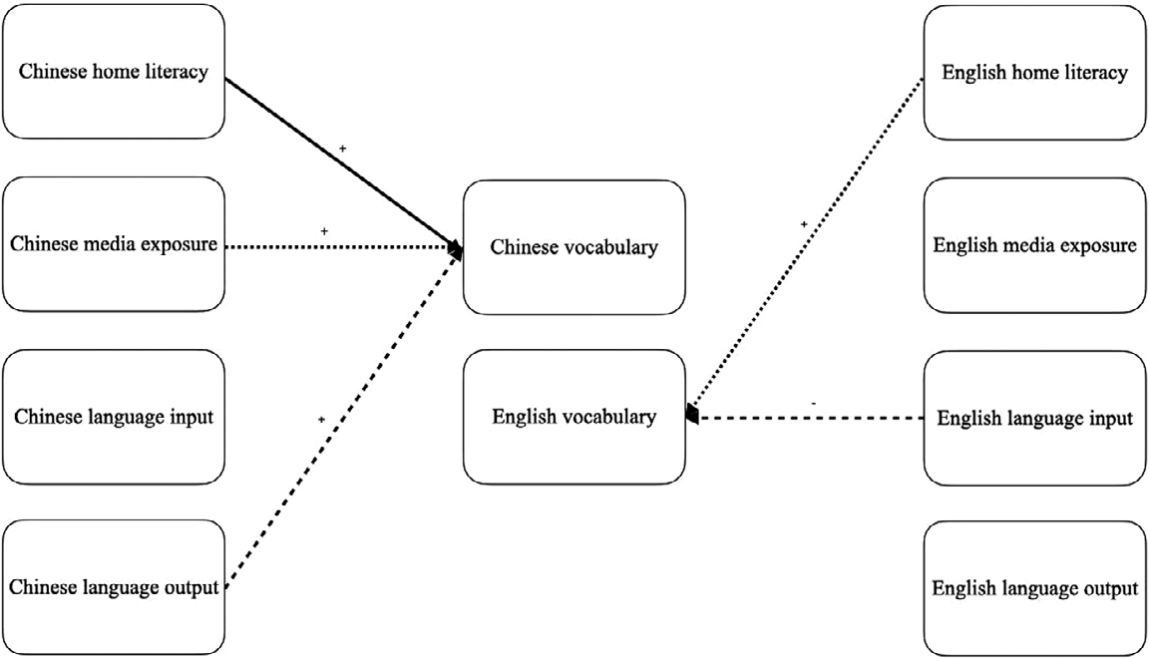
Relationships between Home Literacy Practices, Language Input/Output, and Vocabulary for Chinese American Participants Pre-Pandemic and During the Pandemic. Solid line = Time 1 and Time 2 significant relationship, dotted line = Time 1 significant relationship, dashed line = Time 2 significant relationship, + = positive relationship, − = negative relationship. Vocabulary was measured with Picture Vocabulary and Oral Comprehension. Child age and pre-pandemic vocabulary were controlled. Standardised beta coefficients can be found in the multiple regression tables.

The multiple regression analyses in Table 12 showed that the Time 1 Chinese Home Literacy and Time 1 Chinese Media were positively related to Chinese American DLLs’ Chinese Picture Vocabulary raw score before the pandemic. In contrast, only media exposure was positively associated with Chinese Oral Comprehension before the pandemic. The Chinese home literacy practice component during the pandemic significantly affected the Chinese Picture Vocabulary raw score. Chinese language output was positively related to Chinese Oral Comprehension raw score when controlling for age and pre-pandemic Chinese language scores. On the other hand, as shown in Table 13, English home literacy was positively associated with Time 1 pre-pandemic English oral comprehension. During the pandemic, English language input was negatively associated with Chinese American DLLs’ English picture vocabulary and oral comprehension when controlling for age and pre-pandemic English oral proficiency scores. These relationships are visually represented in Figure 2.

**Table 12.** Multiple regression testing relations of Chinese Home Literacy Practices and Chinese Oral Language Proficiency in Chinese American children (T1 = Time 1 [pre-pandemic], T2 = Time 2 [during pandemic], CHI = Chinese, PV = Woodcock Johnson Picture Vocabulary, OC = Woodcock Johnson Oral Comprehension, R = raw score, HLP = home literacy practices, *B* = unstandardised regression coefficient, *β* = standardised regression coefficient, SE = standard error; *p < .05, ^**^ *p* < .01, ^***^p < .001)

	T1 CHI PV (R)			T1 CHI OC (R)			T2 CHI PV (R)			T2 CHI OC (R)		
Predictors	*B*	*β*	SE	*B*	*β*	SE	*B*	*β*	SE	*B*	*β*	SE
T1 CHI home literacy	1.62*	.24	.64	.07	.04	.20						
T1 CHI media	2.57^**^	.21	1.11	.83*	.25	.34						
T1 CHI input	5.13	.13	6.43	.69	.06	2.03						
T1 CHI output	7.11	.23	4.93	.83	.10	1.52						
T2 CHI home literacy							1.71^***^	.29	.46	.42	.12	.33
T2 CHI media							.59	.12	.37	.51	.19	.29
T2 CHI input							−2.35	−.07	3.47	−2.60	−.13	2.4
T2 CHI output							1.34	.04	3.18	4.81*	.27	2.21
T1 child age	.23^**^	.25	.08	.10^***^	.38	.03						
T2 child age							.11	.11	.08	.21*	.36	.06
T1 CHI PV (R)							.52^***^	.57	.07			
T1 CHI OC (R)										.48*	.27	.20
Constant	−8.78		4.90	−4.83^**^		1.50	1.20		5.46	−12.33^**^		3.91
*R^2^*	.33			.22			.59			.40		
Adjusted *R^2^*	.29			.17			.55			.35		
*F*	8.32^***^			4.55^***^			17.82^***^			7.91^***^		
*N*	89			86			81			80

**Table 13.** Multiple regression testing relations of English Home Literacy Practices and English Oral Language Proficiency in Chinese American participants (T1 = Time 1 [pre-pandemic], T2 = Time 2 [during pandemic], ENG = English, PV = Woodcock Johnson Picture Vocabulary, OC = Woodcock Johnson Oral Comprehension, S = standard score, HLP = home literacy practices, *B* = unstandardised regression coefficient, *β* = standardised regression coefficient, SE = standard error; *p < .05, ^**^ *p* < .01, ^***^p < .001)

	T1 ENG PV (S)			T1 ENG OC (S)			T2 ENG PV (S)			T2 ENG OC (S)		
Predictors	*B*	*β*	SE	B	β	SE	*B*	*β*	SE	*B*	*β*	SE
T1 ENG home literacy	2.65	.18	1.67	3.64*	.26	1.61						
T1 ENG media	2.02	.12	1.84	1.07	.07	1.78						
T1 ENG input	−13.85	−.15	17.42	6.80	.08	16.79						
T1 ENG output	23.30	.32	13.66	−6.68	−.10	13.16						
T2 ENG home literacy							1.38	.10	1.441	2.90	.18	1.78
T2 ENG media							1.37	.16	.92	.25	.02	1.11
T2 ENG input							−23.69*	−.30	10.59	−29.23*	−.31	12.76
T2 ENG output							17.44	.24	9.75	18.66	.21	11.77
T1 ENG PV (S)							.28^**^	.33	.09			
T1 ENG OC (S)										.34^**^	.31	.12
Constant	71.70^***^		4.35	68.91^***^		4.19	67.45^***^		8.69	71.10^***^		10.75
*R^2^*	.10			.06			.20			.21		
Adjusted *R^2^*	.06			.02			.15			.15		
*F*	2.32			1.39			3.67^**^			3.81^**^		
*N*	86			86			82			84

## 10. Discussion

This study investigated the impact of the COVID-19 pandemic on the oral language development of low-income Mexican American and Chinese American DLLs by focusing on the relationships between home literacy practices, language use, and oral language proficiency in both English and their home languages. The findings reveal a complex picture of language development during unprecedented disruption to children’s lives.

### 10.1. Language development and resilience

Despite the significant challenges posed by extended school closures and online learning during the pandemic, Mexican American and Chinese American DLLs demonstrated improved oral proficiency in English and their home language. However, it is crucial to note that the initial levels of oral language proficiency in both languages were significantly below age-normed expectations for both groups. This finding underscores the significant language vulnerabilities faced by low-income DLLs, particularly during periods of significant disruption such as the COVID-19 pandemic. This observation aligns with the Integrative Risk and Resilience Model (Suárez-Orozco et al., 2018), which emphasises the complex interplay of individual, family, and societal factors that influence the development of immigrant children. In this context, the low baseline language skills can be viewed as an existing risk factor for these children. While the observed improvements in language proficiency are encouraging, they highlight the need for ongoing support to mitigate the significant language vulnerabilities these children face.

Furthermore, the observed improvements in both English and home language proficiency suggest that these low-income immigrant DLL children may have demonstrated resilience in the face of adversity. Despite the challenges posed by the pandemic, they could maintain and even enhance their language skills. This resilience may be attributed to many factors, including strong family support, access to community resources, and the children’s inherent strengths and abilities. However, it is essential to acknowledge that the observed improvements may not be sufficient to fully mitigate the potential long-term impacts of the pandemic on the educational trajectories of these children. Continued monitoring and support are crucial to ensure that these children have the opportunity to reach their full academic potential.

### 10.2. Shifting communication patterns and language development

Before the pandemic, parent–child communication within both groups primarily occurred in the home language. However, during the pandemic, a balanced use of English and the home language emerged, which suggested a shift in communication patterns. This finding is significant as it indicates that DLLs’ home language use persisted while their English proficiency continued to develop, even with the disruptions to formal schooling. This aligns with previous research suggesting increased English use in multilingual households as children enter English-medium education (Ke et al., 2023). However, it is crucial to note that this shift in language use may have varied across families depending on factors like parental language proficiency, parental employment status, and access to community resources.

### 10.3. The role of home literacy practices

The finding challenges the assumption that increased English usage in the home environment translates directly to improved English oral proficiency for DLLs. While both groups demonstrated increased English home literacy practices, only English language input demonstrated a significant association with English proficiency. This suggests that the quantity of English exposure, including home literacy activities, may not be the sole determinant of English language development. Instead, the quality of English language input, characterised by engaging interactions, rich vocabulary, and meaningful communication, may be more crucial for promoting English language acquisition. Quality interactions involve responsive communication with frequent turn-taking, where parents actively elicit children’s responses and build upon their contributions. They also include exposing children to diverse and sophisticated words and engaging in contextually relevant and meaningful conversations.

While shared book reading is often regarded as an activity involving high-quality language exchanges (e.g., Dowdall et al., 2020; Whitehurst et al., 1988), its effectiveness can vary depending on the quality of the interactions. Reading aloud with minimal interaction or focusing primarily on letter and word teaching without discussing the story content may represent lower quality shared reading. In contrast, high-quality shared reading is characterised by active engagement, dialogic reading, and child involvement (e.g., Kaderavek et al., 2014). Furthermore, the quality of shared book reading can be influenced by parents’ choice of book and reading practices, such as illustrations, story structure, and opportunity for child engagement (Bergman Deitcher et al., 2019). Parents who utilise strategies that promote quality interactions, such as using open-ended questions and inferential discussions, tend to foster better child language outcomes (e.g., Han & Neuharth-Pritchett, 2014; Marjanovič-Umek et al., 2019). Quality language interactions can also occur outside of traditional home literacy activities, such as through daily routines like cooking, shopping, or engaging storytelling that encourages the children’s active participation.

This finding aligns with the Home Literacy Model (Sénéchal et al., 1998), which emphasises the importance of both formal and informal literacy experiences. While our study included measures of both formal and informal literacy activities, the results suggest that the specific types of English home literacy practices measured in this study might not have been directly related to English language development. Future research should investigate the nature and quality of these practices, such as the impact of specific shared reading and storytelling techniques, including dialogic reading (e.g., Pillinger & Vardy, 2022; Whitehurst et al., 1988), challenging text selection, and child engagement (e.g., Dickinson & Tabors, 2001; Kaderavek et al., 2014). Additionally, investigating English media selections and the nature of parental involvement in children’s learning activities, including helping DLLs learn numbers, letters, and words and helping them with homework, would help uncover what specific literacy activities play a role in shaping low-income immigrant DLLs’ bilingual language proficiency.

Additionally, the limitations of measuring exposure based solely on quantity should be addressed by examining the qualitative aspects of these parent–child interactions. For example, researchers could investigate the complexity of language used by parents during interactions, the extent to which parents engage in responsive and interactive communication, and the types of books and media children are exposed to. Furthermore, the broader concept of English media exposure likely encompasses diverse content, ranging from educational programs focused on literacy to entertainment with relatively less literacy values, which should be further examined in future studies. This heterogeneity of literacy practices calls for more nuanced measurement approaches.

### 10.4. Oral language proficiency and home literacy practices

For Mexican American DLLs, Spanish language use at home remained significantly associated with Spanish oral proficiency throughout the pandemic, which highlights the continued importance of fostering children’s active Spanish language use. This finding suggests that creating opportunities for children to engage in meaningful language experiences in Spanish can significantly contribute to their overall language proficiency. Spanish home literacy practices were not associated with Spanish vocabulary outcomes before or during the pandemic. This finding suggests that Spanish home literacy practices may not account for a substantial proportion of the variance of children’s language development during the pandemic, which requires further investigation, considering potential changes in the nature of these practices during school closures and online learning. For instance, the increased reliance on digital devices may have led to the shift from traditional shared book reading with parent–child dialogic interaction to more passive engagement with electronic books or educational applications, which can potentially influence the relationship between home literacy activities and language learning. Another possible phenomenon is that school closures and the pandemic increased parental stress and time constraints, which might have reduced the frequency and quality of literacy activities at home and further explain the weaker relationship between home literacy and language development. Additionally, other factors, such as individual differences or school learning experiences, may also shape children’s language outcomes. Further, none of the measured English home literacy practices contributed significantly to English oral proficiency during either the pre-pandemic or pandemic periods. This finding suggests that factors other than home literacy practices may be more influential in shaping English language development in this population.

In contrast, Chinese American DLLs experienced an increase in specific English home literacy practices, including English media exposure and parental involvement in English learning, alongside a decrease in Chinese home literacy practices, including oral storytelling and shared book reading. Home literacy practices remained strongly associated with Chinese oral proficiency despite these shifts throughout the pandemic. However, similar to the Mexican American group, no significant relationships were found between English home literacy practices and English oral language proficiency before or during the pandemic. Only English language input, rather than specific home literacy practices, was associated with English oral proficiency during the pandemic. These findings suggest that the values, beliefs, and expectations embedded within different cultural groups regarding language learning and home literacy practices and the unique linguistic characteristics of each language may significantly influence language development.

The non-significant relationships between most measured home literacy practices and English language outcomes warrant further investigation. The findings suggest that (1) the current study might have missed capturing key home literacy practices that significantly influence oral language proficiency, or (2) home literacy practices, while important, may not be the primary contributor to oral language proficiency for DLLs. Other factors, such as quality of school instruction and peer influence, might play a more substantial role. Furthermore, the study’s focus on parent–child interactions may have limited its ability to capture the full range of literacy experiences that influence children’s language development. For instance, interactions with extended family members, teachers, and community members can also provide valuable language learning opportunities. These findings emphasise the need for a more nuanced understanding of the relationship between home, school, and community factors in shaping the language development of DLLs. Further research is necessary to identify specific home literacy practices and broader sociocultural contexts that effectively contribute to developing English oral language proficiency in low-income immigrant DLLs during their early school years.

### 10.5. Limitations and future directions

There are several limitations to the study that could account for the gaps in our results. The historical events associated with the COVID-19 pandemic created unforeseen limitations to the study. Due to the pandemic, the data had to be collected virtually in home settings. While remote data collection has become increasingly common, it is essential to acknowledge that there may be potential limitations compared to in-person assessment. One potential concern is that, as the data was collected virtually for the first time, the participants may have performed worse or better on Zoom. Factors such as distractions, comfort level, and technological limitations could have influenced children’s performance. The novelty of remote testing may also have caused children to experience anxiety or stress, which could have affected their responses. While Wright (2016) found no significant differences between scores on Woodcock-Johnson IV assessments delivered remotely and in person, Thomas and colleagues (2022) suggested potential limitations to consider when testing young children, especially DLLs, over Zoom. For example, it can be challenging to maintain young children’s attention and engagement in a virtual testing environment. Therefore, we implemented several strategies, such as providing technical support, ensuring a comfortable and distraction-free testing environment, and offering breaks to mitigate these potential challenges. However, it is still important to acknowledge that these measures may not have entirely eliminated the potential impact of remote testing on children’s performance. However, given the circumstances of the pandemic and the need to continue data collection, we believe the adaptations we implemented were necessary and appropriate.

Additionally, there may be some gaps in support as children transition into an online learning environment at home. The inequities in learning at home due to various socio-economic factors may be exacerbated by collecting data online. Further, the observed changes in oral language proficiency may be influenced by natural developmental trajectories within the three- to five-year-old range of the participants in this study. This age range was chosen to capture a representative sample of young children attending Head Start programs, typically serving children aged 3–5 years. While this age range enhances the generalisability of our findings to Head Start populations, it may also contribute to increased variability within each DLL group. We controlled for age in our regression models to account for its potential influence on the relationships between home literacy practices and language outcomes. While age was controlled for in the statistical analyses, future research with a narrower age range or longitudinal designs could provide a more nuanced understanding of the specific contributions of age and other factors to language growth in the low-income immigrant DLL populations. Lastly, the study was limited by assessment tools that may not be appropriate for DLLs, especially for Chinese-English DLLs. As the assessment tools used are made for monolingual English-speaking and Spanish-speaking students, the study expects more accurate results with a more suitable test that accounts for our participants’ context.

## 11. Conclusion

The present study provides valuable insights into the language development of low-income Mexican American and Chinese American DLLs during the COVID-19 pandemic. Despite the significant challenges posed by school closures and disruptions to in-person learning, both groups demonstrated improvements in both English and their home languages. This finding highlights the resilience of these children and the importance of continued language support within the home environment. However, the study also underscores the significant language vulnerabilities these children face. Baseline levels of oral language proficiency in both English and their home languages were significantly below age-normed expectations for both groups, which emphasised the need for ongoing support to mitigate the potential long-term impacts of the pandemic on their educational trajectories.

Furthermore, the study revealed a complex relationship between home literacy practices and language development. While increased English language use was observed in both groups, the specific impact of measured home literacy practices on English oral proficiency was limited. These findings suggest that factors beyond traditional home literacy practices, such as the quality of language input and the broader sociocultural context, play a crucial role in shaping the language development of DLLs. Future research should investigate the specific types of home literacy practices that most effectively support the language development of DLLs, particularly in the context of diverse cultural and linguistic backgrounds. Examining the quality of language input within the home environment, including the complexity and diversity of language used, will be crucial for a deeper understanding of these relationships. Additionally, longitudinal research is needed to track the long-term impacts of the pandemic on the language and academic trajectories of these children.

## References

[r1] August, D., & Shanahan, T. (Eds.) (2006). Developing literacy in second-language learners. Erlbaum.

[r2] Ballotpedia. (n.d.). *School responses in California to the coronavirus* *(COVID-19)* *pandemic*. Retrieved July 28, 2025, from https://ballotpedia.org/School_responses_in_California_to_the_coronavirus_(COVID-19)_pandemic

[r3] Bedore, L. M., Peña, E. D., Summers, C. L., Boerger, K. M., Resendiz, M. D., Greene, K., Bohman, T. M., & Gillam, R. B. (2012). The measure matters: Language dominance profiles across measures in Spanish–English bilingual children. Bilingualism: Language and Cognition, 15(3), 616–629. 23565049 10.1017/S1366728912000090 PMC3615884

[r4] Bergman Deitcher, D., Aram, D., & Adar, G. (2019). Book selection for shared reading: Parents’ considerations and researchers’ views. Journal of Early Childhood Literacy, 19(3), 291–315. 10.1177/1468798417718236.

[r5] Bohman, T. M., Bedore, L. M., Peña, E. D., Mendez-Perez, A., & Gillam, R. B. (2010). What you hear and what you say: Language performance in Spanish–English bilinguals. International Journal of Bilingual Education and Bilingualism, 13(3), 325–344. 21731899 10.1080/13670050903342019 PMC3128885

[r7] Burgess, S. R., Hecht, S. A., & Lonigan, C. J. (2002). Relations of the home literacy environment (HLE) to the development of reading-related abilities: A one-year longitudinal study. Reading Research Quarterly, 37(4), 408–426. 10.1598/rrq.37.4.4.

[r8] Bus, A. G., van IJzendoorn, M. H., & Pellegrini, A. D. (1995). Joint book reading makes for success in learning to read: A meta-analysis on intergenerational transmission of literacy. Review of Educational Research, 65(1), 1–21.

[r9] Chernoff, J. J., Keuter, S., Uchikoshi, Y., Quick, H., & Manship, K. (2021). Challenges in assessing California’s diverse dual language learners. American Institute for Research. https://www.air.org/sites/default/files/First-5-DLL-Pilot-Study-Assessment-Challenges-508-Feb-2021.pdf

[r10] Child Trends. (2019, March 07). *Dual language learners. Retrieved August 1, 2022, from* https://www.childtrends.org/indicators/dual-language-learners

[r11] Chung, S., Zhou, Q., Anicama, C., Rivera, C., & Uchikoshi, Y. (2019). Language proficiency, parenting styles, and socioemotional adjustment of young dual language learners. Journal of Cross-Cultural Psychology., 50(7), 896–914. 10.1177/0022022119867394. 31543546 PMC6753939

[r12] Crosson, A. C., & Silverman, R. D. (2021). Impact of COVID-19 on early literacy instruction for emergent bilinguals. Reading Research Quarterly, 57(1), 5–14. 10.1002/rrq.456.

[r13] Davison, M. D., Hammer, C., & Lawrence, F. R. (2011). Associations between preschool language and first grade reading outcomes in bilingual children. Journal of Communication Disorders, 44(4), 444–458. 10.1016/j.jcomdis.2011.02.003. 21477813 PMC3138808

[r14] De Ramírez, R. D., & Shapiro, E. S. (2007). Cross-language relationship between Spanish and English oral reading fluency among Spanish-speaking English language learners in bilingual education classrooms. Psychology in the Schools, 44(8), 795–806. 10.1002/pits.20266.

[r90] Desjardins J. L., Barraza, E. G., & Orozco, J. A. (2019). Age-related changes in speech recognition performance in Spanish-English bilinguals’ first and second languages. Journal of Speech, Language, and Hearing Research, 62(7), 2553–2563. 10.1044/2019_JSLHR-H-18-0435 PMC6808351 31251686

[r15] Dickinson, D. K., & P. O. Tabors (Eds.), (2001). Beginning literacy with language: Young children learning at home and school. Paul H. Brookes Publishing.

[r16] Dickinson, D. K., McCabe, A., Clark–Chiarelli, N. A. N. C. Y., & Wolf, A. (2004). Cross-language transfer of phonological awareness in low-income Spanish and English bilingual preschool children. Applied PsychoLinguistics, 25(3), 323–347. 10.1017/S0142716404001158.

[r17] Dowdall, N., Melendez-Torres, G. J., Murray, L., Gardner, F., Hartford, L., & Cooper, P. J. (2020). Shared picture book Reading interventions for child language development: A systematic review and meta-analysis. Child Development, 91(2), e383–e399. 10.1111/cdev.13225. 30737957

[r18] Durán, L., Wackerle-Hollman, A., Miranda, A., Chávez, C., Pentimonti, J., Zyskind, K., & Rodriguez, M. C. (2022). Spanish and English Oral language growth rates of bilingual Preschoolers: The effect of language of instruction. Learning Disabilities Research & Practice, 37(3), 175–188. 10.1111/ldrp.12287.

[r19] Duursma, E., Romero-Contreras, S., Szuber, A., Proctor, P., Snow, C., August, D., & Calderón, M. (2007). The role of home literacy and language environment on bilinguals’ English and Spanish vocabulary development. Applied PsychoLinguistics, 28(1), 171–190. 10.1017/S0142716406070093.

[r20] Education Week. (2020, March 6). *Map: Coronavirus and School Closures in 2019–2020*. Retrieved August 1, 2022, from https://www.edweek.org/leadership/map-coronavirus-and-school-closures-in-2019-2020/2020/03

[r21] Espinosa, L.M. (2013). Early education for dual language learners: Promoting school readiness and early school success. Migration Policy Institute. https://www.migrationpolicy.org/research/early-education-dual-language-learners-promoting-school-readiness-and-early-school-success

[r22] Farver, J. A. M., Xu, Y., Lonigan, C. J., & Eppe, S. (2013). The home literacy environment and Latino head start children’s emergent literacy skills. Developmental Psychology, 49(4), 775–791. 10.1037/a0028766. 22662767

[r23] Frijters, J. C., Barron, R. W., & Brunello, M. (2000). Direct and mediated influences of home literacy and literacy interest on prereaders’ oral vocabulary and early written language skill. Journal of Educational Psychology, 92(3), 466–477. 10.1037/0022-0663.92.3.466.

[r24] Gámez, P. B., Palermo, F., Perry, J. S., & Galindo, M. (2022). Spanish-English bilingual toddlers’ vocabulary skills: The role of caregiver language input and warmth. Developmental Science, 26(2), e13308. 10.1111/desc.13308. 35913423 PMC10644905

[r25] Giang, I. T. N., & Park, M. (2022). California’s dual language learners: Key characteristics and considerations for early childhood programs. Migration Policy Institute. https://www.migrationpolicy.org/sites/default/files/publications/mpi-nciip_dll-fact-sheet2022_ca-final.pdf

[r26] Gonzalez, J. E., & Uhing, B. M. (2008). Home literacy environments and young Hispanic children’s English and Spanish oral language: A communality analysis. Journal of Early Intervention, 30(2), 116–139. 10.1177/1053815107313858.

[r27] Haft, S. L., Gys, C. L., Bunge, S., Uchikoshi, Y., & Zhou, Q. (2021). Home language environment and executive functions in Mexican American and Chinese American preschoolers in head start. Early Education and Development, 33(4), 608–633. 10.1080/10409289.2021.1912548. 35600115 PMC9119586

[r28] Hammer, C. S., Hoff, E., Uchikoshi, Y., Gillanders, C., Castro, D. C., & Sandilos, L. E. (2014). The language and literacy development of young dual language learners: A critical review. Early Childhood Research Quarterly, 29(4), 715–733. 10.1016/j.ecresq.2014.05.008. 25878395 PMC4394382

[r29] Hammer, C. S., Lawrence, F. R., & Miccio, A. W. (2007). Bilingual children’s language abilities and early reading outcomes in head start and kindergarten. Language, Speech, and Hearing Services in Schools, 38(3), 237–248. 10.1044/0161-1461(2007/025. 17625050 PMC4590989

[r30] Hammer, C. S., Miccio, A. W., & Wagstaff, D. A. (2003). Home literacy experiences and their relationship to bilingual preschoolers’ developing English literacy abilities: An initial investigation. Language Speech and Hearing Services in Schools, 34, 20–30. 10.1044/0161-1461(2003/003. PMC4551497 26321778

[r31] Han, J., & Neuharth-Pritchett, S. (2014). Parents’ interactions with preschoolers during shared book reading: Three strategies for promoting quality interactions. Childhood Education, 90(1), 54–60. 10.1080/00094056.2014.872516.

[r33] Hoff, E. (2013). Interpreting the early language trajectories of children from low SES and language minority homes: Implications for closing achievement gaps. Developmental Psychology, 49(1), 4–14. 10.1037/a0027238. 22329382 PMC4061698

[r34] Hood, M., Conlon, E., & Andrews, G. (2008). Preschool home literacy practices and children’s literacy development: A longitudinal analysis. Journal of Educational Psychology, 100(2), 252–271. 10.1037/0022-0663.100.2.252.

[r35] Howard, E. R., Páez, M. M., August, D. L., Barr, C. D., Kenyon, D., & Malabonga, V. (2014). The importance of SES, home and school language and literacy practices, and oral vocabulary in bilingual children’s English reading development. Bilingual Research Journal, 37(2), 120–141. 10.1080/15235882.2014.934485.

[r91] Im, C. (2025). Chinese in the U.S. fact sheet. Pew Research Center. Retrieved July 28, 2025, from https://www.pewresearch.org/social-trends/fact-sheet/asian-americans-chinese-in-theu-s/

[r36] Inoue, T., Georgiou, G. K., Parrila, R., & Kirby, J. R. (2018). Examining an extended home literacy model: The mediating roles of emergent literacy skills and Reading fluency. Scientific Studies of Reading, 22(4), 273–288. 10.1080/10888438.2018.1435663.

[r37] Kaderavek, J. N., Pentimonti, J. M., & Justice, L. M. (2014). Children with communication impairments: Caregivers’ and teachers’ shared book-reading quality and children’s level of engagement. Child Language Teaching and Therapy, 30(3), 289–302. 10.1177/0265659013513812.

[r38] Ke, S., Xia, Y., & Zhang, J. (2023). What really matters in early bilingual and biliteracy acquisition?: Home language and literacy input in Chinese heritage language learners. Researching and Teaching Chinese as a Foreign Language, 4(1), 73–96. 10.1558/rtcfl.24922.

[r39] Kieffer, M. J. (2012). Early oral language and later reading development in Spanish-speaking English language learners: Evidence from a nine-year longitudinal study. Journal of Applied Developmental Psychology, 33(3), 146–157. 10.1016/j.appdev.2012.02.003.

[r40] Kuhfeld, M., Soland, J., Tarasawa, B., Johnson, A., Ruzek, E., & Liu, J. (2020). Projecting the potential impact of COVID-19 school closures on academic achievement. Educational Researcher, 49(8), 549–565. 10.3102/0013189X20965918.

[r41] Lervåg, A., Hulme, C., & Melby-Lervåg, M. (2018). Unpicking the developmental relationship between oral language skills and reading comprehension: It’s simple but complex. Child Development, 89(5), 1821–1838. 10.1111/cdev.12861. 28605008

[r42] Li, L., & Tan, C. L. (2016). Home literacy environment and its influence on Singaporean children’s Chinese oral and written language abilities. Early Childhood Education Journal, 44(4), 381–387. 10.1007/s10643-015-0723-4.

[r43] Lin, N. T., Molgaard, M., Wishard Guerra, A., & Cohen, S. (2023). Young children and families’ home literacy and technology practices before and during COVID-19. Journal of Early Childhood Research, 21(3), 341–354. 10.1177/1476718X231164132.

[r45] Luo, R., Pace, A., Levine, D., Iglesias, A., de Villiers, J., Golinkoff, R. M., Wilson, M. S., & Hirsh-Pasek, K. (2021). Home literacy environment and existing knowledge mediate the link between socioeconomic status and language learning skills in dual language learners. Early Childhood Research Quarterly, 55, 1–14. 10.1016/j.ecresq.2020.10.007.

[r46] Mak, E., Nichiporuk Vanni, N., Yang, X., Lara, M., Zhou, Q., & Uchikoshi, Y. (2023). Parental perceptions of bilingualism and home language vocabulary: Young bilingual children from low-income immigrant Mexican American and Chinese American families. Frontiers in Psychology, 14, 1059298. 10.3389/fpsyg.2023.1059298. 36818097 PMC9928566

[r47] Marjanovič-Umek, L., Hacin, K., & Fekonja, U. (2019). The quality of mother–child shared reading: Its relations to child’s storytelling and home literacy environment. Early Child Development and Care, 189(7), 1135–1146. 10.1080/03004430.2017.1369975.

[r49] McGrew, K. S., LaForte, E. M., & Schrank, F. A. (2014). Woodcock-Johnson IV technical manual. Riverside Insights.

[r50] Migration Policy Institute. (2021). U.S. young children (ages 0 to 8) by dual language learner status: National and state sociodemographic and family profiles. Migration Policy Institute. https://www.migrationpolicy.org/sites/default/files/datahub/us-dll-children-profiles-age0-8_final.xlsx

[r51] National Academies of Sciences, Engineering, and Medicine. (2017). Promoting the educational success of children and youth learning English: Promising futures. The National Academies Press. 10.17226/24677.

[r77] National Center for Education Statistics. (2024). *English language learners in public schools.* U.S. Department of Education, Institute of Education Sciences. Retrieved July 28, 2025, from https://nces.ed.gov/programs/coe/indicator/cgf/english-learners-in-public-schools

[r52] Parolin, Z., & Wimer, C. (2020). Forecasting estimates of poverty during the COVID-19 crisis. Poverty and Social Policy Brief, 4(8), 1–18. https://povertycenter.columbia.edu/sites/povertycenter.columbia.edu/files/content/Publications/Forecasting-Poverty-Estimates-COVID19-CPSP-2020.pdf

[r53] Peña, E. D., Gutierrez-Clellen, V. F., Iglesias, A., Goldstein, B. A., & Bedore, L. M. (2014). Bilingual English Spanish assessment (BESA). AR Clinical Publications.

[r54] Petscher, Y., Cabell, S. Q., Catts, H. W., Compton, D. L., Foorman, B. R., Hart, S. A., Lonigan, C. J., Philips, B. M., Schatschneider, C., Steacy, L. M., Terry, N. P., & Wagner, R. K. (2020). How the science of reading informs 21st-century education. Reading Research Quarterly, 55(S1), S267–S282. 10.1002/rrq.352. 34007089 PMC8128160

[r56] Pillinger, C., & Vardy, E. J. (2022). The story so far: A systematic review of the dialogic reading literature. Journal of Research in Reading, 45(4), 533–548. 10.1111/1467-9817.12407.

[r57] Poulain, T., Meigen, C., Sobek, C., Ober, P., Igel, U., Körner, A., Kiess, W., & Vogel, M. (2021). Loss of childcare and classroom teaching during the Covid-19-related lockdown in spring 2020: A longitudinal study on consequences on leisure behavior and schoolwork at home. PLoS One, 16(3), e0247949. 10.1371/journal.pone.0247949. 33651851 PMC7924794

[r93] Quiroz, B. G., Snow, C. E., & Zhao, J. (2010). Vocabulary skills of Spanish-English bilinguals: Impact of mother-child language interactions and home language and literacy support. International Journal of Bilingualism, 14(4), 379–399. 10.1177/1367006910370919

[r58] Read, K., Gaffney, G., Chen, A., & Imran, A. (2021). The impact of COVID-19 on families’ home literacy practices with young children. Early Childhood Education Journal, 50(8), 1429–1438. 10.1007/s10643-021-01270-6. 34629842 PMC8488072

[r59] RStudio Team (2021). RStudio: Integrated development environment for R. [Computer software] RStudio, PBC. http://www.rstudio.com/

[r60] Rydland, V., & Grøver, V. (2021). Language use, home literacy environment, and demography: Predicting vocabulary skills among diverse young dual language learners in Norway. Journal of Child Language, 48(4), 717–736. 10.1017/S0305000920000495. 33023680

[r61] Scarborough, H. S., & Dobrich, W. (1994). Another look at parent-preschooler bookreading: How naked is the emperor?: A response to Lonigan (1994) and dunning, Mason, and Stewart (1994). Developmental review, 14(3), 340–347. 10.1006/drev.1994.1013

[r62] Schrank, F. A., Mather, N., & McGrew, K. S. (2014). Woodcock-Johnson IV tests of Oral language. Riverside Insights.

[r63] Schrank, F. A., McGrew, K. S., Ruef, M. L., Alvarado, C. G., Muñoz-Sandoval, A. F., & Woodcock, R. W. (2005). Overview and technical supplement (Batería III Woodcock-Muñoz assessment service bulletin no. 1). Riverside Insights.

[r64] Sénéchal, M. (2006). Testing the home literacy model: Parent involvement in kindergarten is differentially related to grade 4 reading comprehension, fluency, spelling, and reading for pleasure. Scientific Studies of Reading, 10(1), 59–87. 10.1207/s1532799xssr1001_4.

[r65] Sénéchal, M., & Lefevre, J. A. (2014). Continuity and change in the home literacy environment as predictors of growth in vocabulary and reading. Child Development, 85(4), 1552–1568. 10.1111/cdev.12222. 24467656

[r94] Sénéchal, M., & LeFevre, J. A. (2002). Parental involvement in the development of children’s reading skill: A five-year longitudinal study. Child Development, 73(2), 445–460. 10.1111/1467-8624.00417 11949902

[r66] Sénéchal, M., LeFevre, J. A., Hudson, E., & Lawson, E. P. (1996). Knowledge of storybooks as a predictor of young children’s vocabulary. Journal of Educational Psychology, 88(3), 520–536. 10.1037/0022-0663.88.3.520.

[r67] Sénéchal, M., Lefevre, J.-A., Thomas, E. M., & Daley, K. E. (1998). Differential effects of home literacy experiences on the development of oral and written language. Reading Research Quarterly, 33(1), 96–116. 10.1598/rrq.33.1.5.

[r68] Shaffer, D. R., & Kipp, K. (2013). Developmental psychology: Childhood and adolescence. Cengage Learning.

[r69] Shanahan, T., & Lonigan, C. J. (2010). The National Early Literacy Panel: A summary of the process and the report. Educational Researcher, 39(4), 279–285. 10.3102/0013189X10369172. PMC3268038 22294803

[r70] Stites, M. L., Sonneschein, S., & Galczyk, S. H. (2021). Preschool parents’ views of distance learning during COVID-19. Early Education and Development, 32(7), 923–939. 10.1080/10409289.2021.1930936.

[r71] Storch, S. A., & Whitehurst, G. J. (2002). Oral language and code-related precursors to reading: Evidence from a longitudinal structural model. Developmental Psychology, 38(6), 934–947. 10.1037/0012-1649.38.6.934. 12428705

[r72] Suárez-Orozco, C., Motti-Stefanidi, F., Marks, A., & Katsiaficas, D. (2018). An integrative risk and resilience model for understanding the adaptation of immigrant-origin children and youth. American Psychologist, 73(6), 781–796. 10.1037/amp0000265. 30188166

[r73] Sun, H., Tan, J., & Chen, W. (2023). COVID-19 and bilingual children’s home language environment: Digital media, socioeconomic status, and language status. Frontiers in Psychology, 14, 1115108. 10.3389/fpsyg.2023.1115108. 37397337 PMC10313223

[r74] The NICHD Early Child Care Research Network. (2005). Child care and child development: Results from the NICHD study of early child care and youth development. The Guilford Press.

[r75] Thomas, L. J. G., Lee, M. G., Todd, C. S., Lynch, K., Loeb, S., McConnell, S., & Carlis, L. (2022). Navigating virtual delivery of assessments for head start children during the COVID-19 pandemic. Journal of Early Intervention, 44(2), 151–167. 10.1177/10538151221085942.

[r76] Torppa, M., Tolvanen, A., Poikkeus, A.-M., Eklund, K., Lerkkanen, M.-K., Leskinen, E., & Lyytinen, H. (2007). Reading development subtypes and their early characteristics. Annals of Dyslexia, 57(1), 3–32. 10.1007/s11881-007-0003-0. 17849214

[r78] Uchikoshi, Y. (2013). Predictors of English reading comprehension: Cantonese-speaking English language learners in the US. Reading and Writing., 26(6), 913–939. 10.1007/s11145-012-9398-z.

[r79] Umansky, I. (2020). COVID-19’s impact on English learner students: Possible policy responses. Policy Analysis for California Education Newsroom Post. https://edpolicyinca.org/newsroom/covid-19s-impact-english-learner-students

[r80] Unsworth, S., Van Den Akker, M., & Van Dijk, C. (2024). The impact of the Covid-19 pandemic on multilingual families in the Netherlands. Journal of Child Language, 52(2), 312–333. 10.1017/S0305000923000715. 38239034

[r81] Wagley, N., Marks, R. A., Bedore, L. M., & Kovelman, I. (2022). Contributions of bilingual home environment and language proficiency on children’s Spanish–English reading outcomes. Child Development, 93(4), 881–899. 10.1111/cdev.13748. 35289947 PMC9619386

[r82] Weigel, D. J., Martin, S. S., & Bennett, K. K. (2006). Contributions of the home literacy environment to preschool-aged children’s emerging literacy and language skills. Early Child Development and Care, 176(3–4), 357–378. 10.1080/03004430500063747.

[r83] Wendling, B. J., Mather, N., & Schrank, F. A. (2019). Examiner’s manual (Bateria IV Woodcock-Muñoz: Pruebas de habilidades cognitivas). Riverside Insights: Riverside Insights.

[r84] Wheeler, D. L., & Hill, J. C. (2021). The impact of COVID-19 on early childhood reading practices. Journal of Early Childhood Literacy, 24(1), 96–115. 10.1177/14687984211044187.

[r85] Whitehurst, G. J., Falco, F. L., Lonigan, C. J., Fischel, J. E., DeBaryshe, B. D., Valdez-Menchaca, M. C., & Caulfield, M. (1988). Accelerating language development through picture book reading. Developmental Psychology, 24(4), 552–559. 10.1037/0012-1649.24.4.552.

[r86] Whitehurst, G. J., & Lonigan, C. J. (2001). Emergent literacy: Development from prereaders to readers. In S. B. Neuman & D. K. Dickensen (Eds.), Handbook of early literacy research (pp. 11–29). Guilford Press.

[r87] Wright, A. J. (2016). Equivalence of remote, online administration and traditional, face-to-face administration of the Woodcock-Johnson IV cognitive and achievement tests [White Paper]. Presence Learning. https://presence.com/wp-content/uploads/2016/09/WJ-IV_Online_Remote_whitepaper_FINAL.pdf

[r88] Zhang, D., & Koda, K. (2011). Home literacy environment and word knowledge development: A study of young learners of Chinese as a heritage language. Bilingual Research Journal, 34(1), 4–18. 10.1080/15235882.2011.568591.

[r89] Zhang, S. Z., Inoue, T., Shu, H., & Georgiou, G. K. (2020). How does home literacy environment influence reading comprehension in Chinese? Evidence from a 3-year longitudinal study. Reading and Writing, 33(7), 1745–1767. 10.1007/s11145-019-09991-2.

